# A distractions capture tool for cardiac surgery and lung transplantation: impact on outcomes

**DOI:** 10.1186/s13019-022-02065-5

**Published:** 2023-01-23

**Authors:** James Arkley, Lay Ping Ong, Niranjan Gunaratnam, Tanveer Butt, Stephen Charles Clark

**Affiliations:** 1 Department of Cardiothoracic Surgery, Freeman Hospital, Newcastle Upon Tyne, UK; 2 Department of Cardiothoracic Surgery, James Cook University Hospital, Middlesbrough, UK; 3 Institute of Transplantation, Freeman Hospital, Newcastle Upon Tyne, UK

**Keywords:** Cardiac surgery, Lung transplantation, Surgical distractions, Patient outcomes

## Abstract

**Objectives:**

Surgical distractions are associated with worse patient outcomes. Lung transplantation and cardiac surgery’s multi-disciplinary nature, and their inherent complexities render them more vulnerable to distractions. We aim to use a novel distractions capture tool to evaluate the severity of distractions during cardiac surgery (CS) and lung transplantation (LTx) and assess its impact on post-operative complications.

**Methods:**

A prospective ‘blinded’ study was undertaken by direct observation of distractions during CS and LTx. Events were identified using the Imperial College Error Capture tool (ICECAP). Number and severity of distractions were correlated with post-operative outcomes (ICU & hospital stay, bleeding and anastomotic complications).

**Results:**

In LTx, we observed 2059 distractions within 287 h across 41 surgeries. In CS, we observed 1089 distractions within 192 h across 62 surgeries. Surgeons were consciously aware of 19.2% (LTx) and 21.3% (CS) of recorded events. Distractions consisted of procedure-independent pressures (61% LTx vs 56% CS), equipment problems (15% LTx vs 23%CS), communication (12% LTx vs 12% CS), technical problems or patient safety concerns (12% LTx vs 9% CS). In CS, 91% of procedure-independent pressures were non-operative distractions whilst LTx recorded 83%. Staff absences at a critical moment of surgery were recorded at 9% (LTx) and 7% (CS). The number and severity of distractions correlated with bleeding (CS *p* < 0.001, LTx *p* < 0.01), prolonged ICU stay (CS *p* = 0.002, LTx *p* = 0.002), hospital stay (CS *p* < 0.001) and anastomotic complications(LTx *p* < 0.03).

**Conclusions:**

ICECAP as a novel surgical distractions capture tool was effective & applicable to both elective cardiac and urgent transplant surgeries. Surgeons were unaware of a large number of distractions & interruptions. Distractions were associated with longer ICU stay and higher rate of bleeding.

**Supplementary Information:**

The online version contains supplementary material available at 10.1186/s13019-022-02065-5.

## Introduction

Interruptions and non-operative distractions lead to surgical errors and are associated with worse patient outcomes [1–3]. Lung transplantation and cardiac surgery’s multi-disciplinary nature, and their inherent complexities render them more vulnerable to distractions [2]. Distractions during surgery are often associated with greater intra-operative stress and perceived workload, thus reducing the medical team’s efficiency and resulting in unwanted errors [4–6]. Errors lead to adverse patient outcomes and additionally places a huge burden on the National Health Service’s (NHS) resources with an estimated cost of around £1bn per year due to additional hospital days alone [7]. Therefore, an improvement in surgical work-flow via a reduction in distractions is paramount.

Firstly, we must be able to identify, quantify and correlate these distractions with patient outcomes before any credible intervention could be undertaken. Although surgical distractions are part of the applied model of human factors analysis of the operating theatre work-flow [1, 2, 8], distractions are difficult to identify, capture and quantify. Other specialties such as urology and laparoscopic surgery have used various prospective observational methods, in order to study the surgical work-flow and the impact of distractions within the operating theatre [9, 10]. However, these methods were limited to surgeries of significantly shorter durations than cardiothoracic surgery.

A novel surgical capture tool (Imperial College Error Capture tool, ICECAP) has been designed and validated within vascular and endovascular surgical theatres [3]; a cousin specialty to cardiothoracic surgery with many similar inherent complexities within the operating theatre work-flow and team structure (Additional file 1). Therefore, we utilized the ICECAP in our study to evaluate the frequency and severity of distraction events during cardiac surgery (CS) and lung transplantation (LTx), and assess the impact of distractions on post-operative complications. Ultimately, we aim to systematically reduce distractions and improve our theatre work-flow in order to better our patient outcomes.

## Materials and methods

### Patients and study design

This observational prospective study was undertaken by direct observation of distractions and interruption events during elective cardiac surgeries and urgent lung transplantations, respectively. We reviewed all adult patients who underwent urgent lung transplantation (LTx) and elective cardiac surgery (CS) in Freeman Hospital, Newcastle-upon-Tyne, UK from August 2011 to 2014. All patients under 18 years old and re-transplants were excluded. Emergency cardiac surgeries and extensive aorto-vascular surgeries were excluded.

**Fig. 1:**
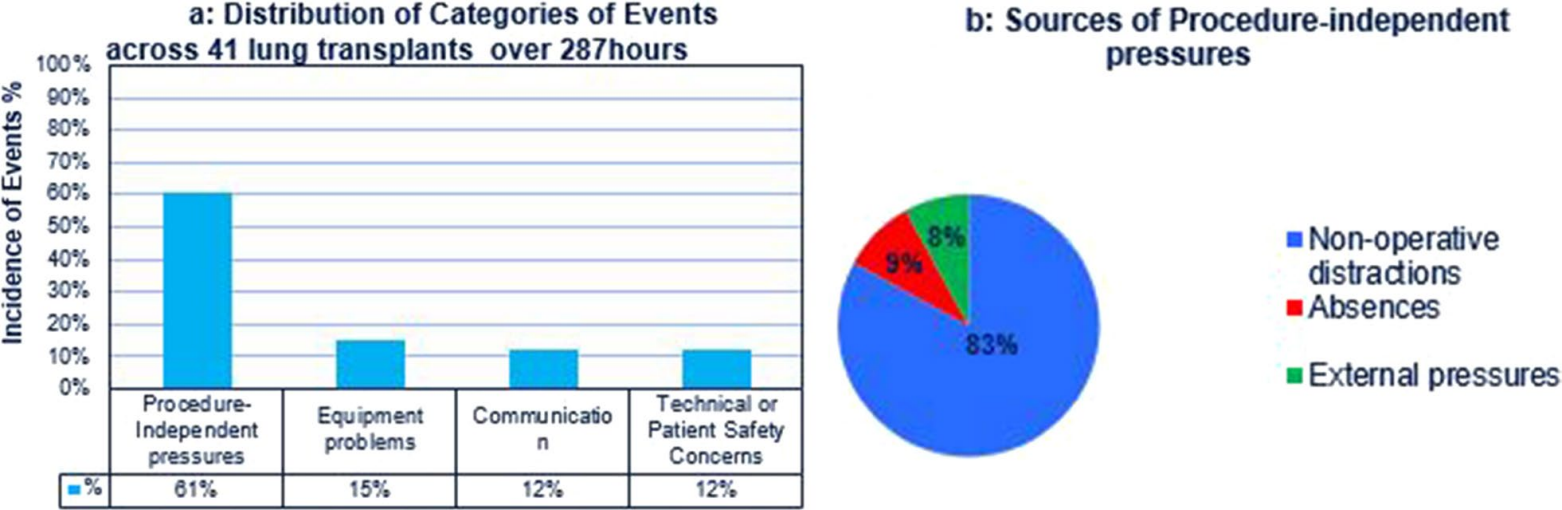
**a** Distribution of ICECAP Categories of Distractions across 41 Lung Transplantations over 287 h **b** Different sources of Procedure-independent Pressures within Lung Transplantation

Events were defined and identified using the Imperial College Error Capture tool (ICECAP) [3], Fig. 1a and b. The entire theatre team (surgeon, surgical assistant, anaesthetists, anaesthetic nurse, scrub team) were ‘blinded’ to study; they were not informed that their work-flow in the operating theatre was being studied. For the duration of this study, an observer is trained to capture events using ICECAP Tool and he/she is present at every case under study. The primary outcome is the number and severity of the distraction events. Secondary outcomes include the correlation of the number and the severity of distraction events with post-operative complications such as ICU stay, total hospital stays, pleural space infections, re-operations for bleeding and anastomotic complications. These outcomes were chosen because they are surrogate markers for the level of mental focus at executing the task at hand.

We also examined clinical variables potentially related to peri-operative risk, such as patients’ age, pre-transplant disease, underlying cardiac pathology, gender, duration of cardiopulmonary bypass (CPB), ischaemic time and Euroscore. Departmental ICU model of care or approach towards cardiac surgery and lung transplantation had not changed dramatically over this time period. The Freeman Hospital Cardiothoracic Cardiac Surgery and Transplant Unit patient databases were utilised for this study. Any variables being examined not contained within the database were collected retrospectively from patient’s electronic or medical records. This observational study was approved by the Research & Development Department, Newcastle Upon Tyne Hospital Trust. Ethical approval was not required as this was an observational study rating operating team performance and no identifiable patient data were collected.

### ICECAP tool

The ICECAP tool was extensively validated based on vascular surgery [3] and was deemed by our team to be highly translatable to cardiac surgery and lung transplantation, due to inherent similarities in surgical complexities, high peri-operative risks, surgical stress and theatre workload. The ICECAP tool was shown to be very effective at capturing and categorizing the number and severity of distraction events [3]. ICECAP Record Sheets and instructions for use were documented within Fig. 1a and b. Each observer was trained to use the ICECAP capture tool. Observers were normally positioned so that they could view and listen to all of the theatre team members; usually at the back of the operating theatre. They were also free-moving during the procedure without obstructing the theatre team. The trained observers were all medical professionals that have been embedded within the theatre team prior to the study. This reduced the likelihood that their presence in theatre would itself be a source of distraction.

From the time of the first skin incision to skin closure, each distraction and error event was recorded. Based on the ICECAP tool, any error event was graded using a 0–5-point scale according to the procedural delay caused and the ease of its resolution [3]. For delay, the error was graded as 0 for those that did not have an effect on the procedural flow, 1 for those with a mild effect on procedural flow and no impact on key tasks, 3 for a moderate impact on procedural flow and 5 for a severe impact on procedural flow with resulting major delays [3]. For ease of resolution, a score of 0 was assigned to errors that required no resolution, 1 for those which were easy to resolve using simple measures, 3 for those that were moderately difficult to resolve requiring the involvement of multiple team members and 5 for those that were almost impossible to resolve requiring involvement of multiple team members [3]. This unique scoring system allows the quantification of the severity of each distraction. At the end of every observed surgery, there would be a team recall exercise. All team members (surgeons, anaesthesiologists, perfusionists, and nurses) were asked to recall errors, causes of delay, interruptions to the flow of the operation and any events that could have resulted in patient harm. The team members’ recall was then correlated with the events recorded by the ICECAP capture tool.

### Statistical analyses

Data were expressed as mean ± one standard deviation (SD) or median with intra-quartile range (IQR). For continuous data, Student’s *t*-test and analysis of variance (ANOVA) were used to determine differences between groups. Chi-squared test was used for categorical variables. A *p* value less than 0.05 was considered significant. Errors recorded on the ICECAP record were entered onto a database (Microsoft Excel) and categorised accordingly. The number and severity of distractions were correlated with post-operative outcomes such as ICU stay, total hospital stay, pleural space infections, re-operations for bleeding and anastomotic complications. Pearson correlation coefficient was used to determine positive or negative correlations, respectively. Statistical analyses were performed using GraphPad Prism 6 software packages.

## Results

### Lung transplantation

During this study, 41 different lung transplantations were observed over a total of 287 h. There were 36 bilateral and 5 single lung transplants; the respective peri-operative characteristics were documented within Table 1. With ICECAP, a total of 2059 interrupting or distracting events were recorded. Following the team recall exercise, we observed that the main operating surgeons were consciously aware of only 19.2% of recorded events during the operation. Based on ICECAP, we demonstrated that 61% were due to procedure-independent pressures, 15% were due to equipment problems, 12% were secondary to communication issues (misunderstandings or inability to hear each other) and the remainder were associated with technical problems or patient safety concerns, Fig. 1a. 83% of procedure-independent pressures were non-operative distractions, Fig. 1b. The 2 most common causes of non-operative distractions were ringing telephones and patient unrelated discussions. 9% were caused by staff absences at a critical moment of surgery.

**Table 1:** Types of Lung Transplantation, intra-operative distractions and post-operative complications

Peri-operative characteristics (Total cases, n = 41)	
Duration of observed surgery (hours)	287
Types of lung transplantation	2 single lung transplantation 39 bilateral lung transplantation
Pre-transplant diagnoses (*n*)	18—emphysema, 20—Cystic Fibrosis, 3—Interstitial Pulmonary Fibrosis
Gender	20 males, 21 females
Total cardiopulmonary bypass (minutes)	232 ± 42
Total ischaemic time (minutes)	267 ± 52
Total number of events (*n*)	2059
Number of events per hour	7.17
Number of events per case	50.2

ICECAP types of distractions	Number of events (n)	Severity
Equipment	309	3.9
Procedure-independent	1256	2.7
Communication	247	4.1
Technical	205	3.9
Safety	42	3.5

Post-operative complications	Correlation coefficient	*p* value
Re-operation for bleeding	0.82	< 0.01
ICU stay	0.76	0.002
Hospital stay	0.51	0.08
Pleural space infection	0.49	0.2
Anastomotic complications	0.78	< 0.03

### Cardiac surgery

**Fig. 2:**
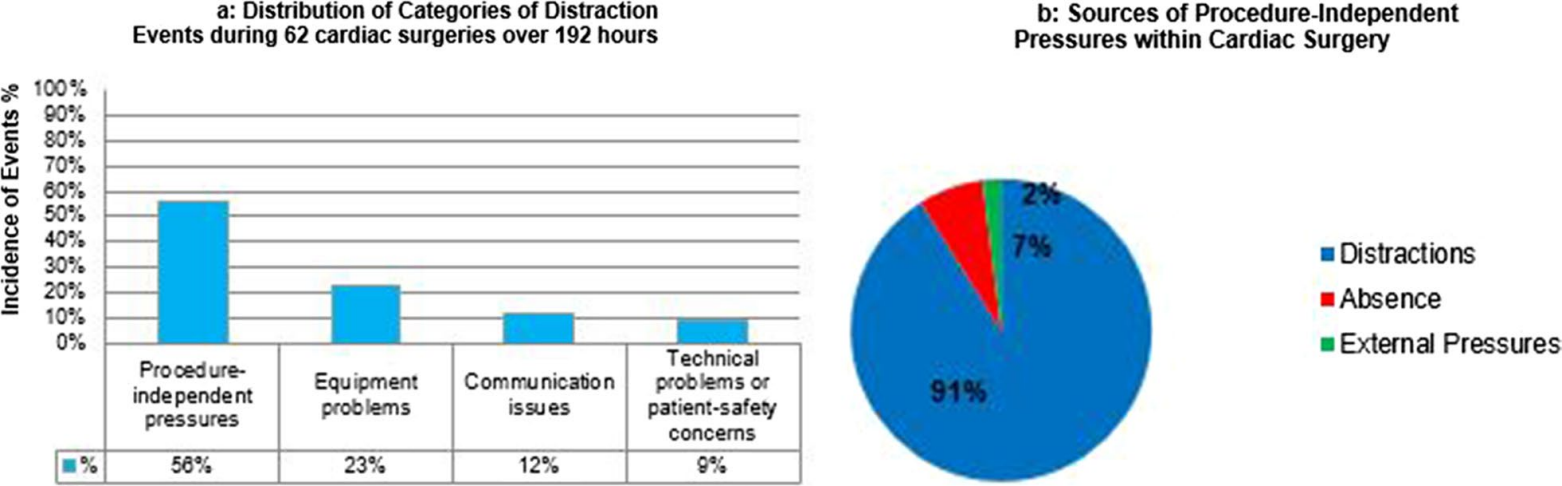
**a** Distribution of ICECAP Categories of Distractions across 62 different Cardiac Surgeries over 192 h **b** Different sources of Procedure-independent Pressures within Cardiac Surgery

As for cardiac surgery, 192 h of surgery were observed across 62 different surgeries; peri-operative characteristics documented within Table 2. There were a total of 1089 distractions. Surgeons were consciously aware of only 21.3% of recorded events whilst operating. Based on ICECAP categorization, 56% were due to procedure-independent pressures, 23% were due to equipment problems, 12% were secondary to communication issues (misunderstandings or inability to hear each other) and the remainder was associated with technical problems or patient safety concerns, Fig. 2a. 91% of procedure-independent pressures were non-operative distractions, Fig. 2b. Similar to lung transplantation, the most common causes of non-operative distractions were ringing telephones and non-patient care-related discussions. 7% were caused by staff absences at a critical moment of surgery.

**Table 2:** Types of Cardiac Surgery, intra-operative distractions and post-operative complications

Peri-operative characteristics (Total cases, n = 62)	
Duration of observed surgery (hours)	192
Types of cardiac surgery *(n)*	32 Coronary Artery Bypass Grafts (CABG), 11 Aortic Valve Replacement (AVR), 19 Mitral Valve Replacement/Repair (MVR)
Gender	35 male, 27 female
total cardiopulmonary bypass (minutes)	83.6 ± 31.2
Total cross clamp time (minutes)	58 ± 18
Elective versus urgent surgery	78% versus 22%
Mean euroscore of observed surgery	5.8
Total number of events (*n*)	1089
Number of events per hour	5.67
Number of events per case	17.6

Types of distractions	Number	Severity
Equipment	250	4.2
Procedure independent	610	3.5
Communication	131	3.9
Technical	88	4.1
Safety	10	3.1
Patient	–	–

Post-operative complications	Correlation coefficient	*p* value
Re-operation for bleeding	0.81	< 0.001
ICU stay	0.76	0.002
Hospital stay	0.85	< 0.001
Infection	0.52	0.527

### Complications

For lung transplantation, we observed that the number and severity of distractions significantly correlated with re-operation for bleeding *(correlation coefficient* = *0.82, p* < *0.01),* anastomotic complications *(correlation coefficient* = *0.78, p* < *0.03)* and prolonged ICU stay *(correlation coefficient* = *0.76, p* = *0.002).* However, there were no statistically significant correlations with pleural space infections or total hospital stay, Table 1. As for cardiac surgery, the number and severity of distractions were significantly correlated with re-operation for bleeding *(correlation coefficient* = *0.81, p* < *0.001),* prolonged ICU stay *(correlation coefficient* = *0.76, p* = *0.002)* and total hospital stay *(correlation coefficient* = *0.85, p* < *0.001)* but not with post-operative infections, Table 2.

## Discussion

### ICECAP tool

Since the advent of the WHO surgical checklist, there has been an increasing focus and effort on improving patient safety within the operating theatre. Although the WHO surgical checklist has reduced many adverse events due to human factors, it does not confer any insights into the surgical work-flow whereby system-based errors still occur. Distractions, with its subsequent disruptions in surgical work-flow have been associated with greater surgical errors and the concomitant burden on NHS resources [1, 7]. In order to deliver successful, highly complex surgeries consistently, we undeniably require a high-performing theatre team with optimal team dynamics that would be reliant upon an interplay of positive chemistry, effective communication and familiarity. Unwanted distractions are intimately linked to surgical stressors that affect the theatre team dynamics and consequently lead to more errors; both minor and major [4–6].

Studies within cardiac surgery and other specialties have used various prospective observational methods to study the surgical work-flow and the impact of distractions within the operating theatre [9–11]. Our distractions capture tool (ICECAP) not only allows for the real-time observation, categorization and capture of the number and severity of distraction events, but it also includes immediate post-operative surgical team feedback and interviews. Additionally, ICECAP was extensively validated. In vascular surgery that shares strong similarities to cardiac surgery. Both specialties involve high-risk, complex operations with similar patient demographics and often involve the interactions of multiple teams (interventional radiologists in vascular surgery). Distraction capture tools documented within other surgical specialties [9, 10, 12] were not deemed to be adequately transferable to cardiac surgery, as the surgeries were less complex, such as hernia repairs and small urological interventions. These surgeries usually only occupy the theatre team for an hour; whilst cardiac surgery and lung transplantation normally occupy the theatre team for 4 h or more.

The usage of a validated distraction capture tool allowed the rapid translation into the operating room, thus obviating the need to have laboratory simulation studies as suggested by Mentis et al. [5]. To investigate the applicability of the capture tool on both elective and emergencies, we chose elective cardiac surgery and emergency lung transplantation. Lung transplantation was used specifically as a comparison because our unit has a relatively high number of lung transplantations per annum. Given this high and consistent level of activity, it seemed more appropriate to capture this rather than cardiac emergencies. In addition, the communication within lung transplantation with the coordination with the donor and recipient unit adds another layer of complexity of human factors that may not occur with cardiac emergencies. Due to the availability of transplantation within our unit, it proved to be a brilliant opportunity for this study.

### Distractions

The ICECAP tool was developed in order to refine the process of error capture, reporting, categorisation and resolution [3]. The ICECAP capture tool encompassed all categories of human factors being studied during the surgical work-flow. Similar to other studies, the categorization of the types of distractions broadly falls into equipment failure, communication, technical faults, safety checks and patient-specific events [11]. The categorization of the types of distraction events is invaluable as it enables a ‘root cause analysis’, thus subsequently allowing the development of a systematic solution to distraction events. Of note, it is often the series of minor distractions events that eventually lead to a major error as highlighted by Martinez et al. [13]. We observed that the most common type of distraction is non-patient-related communications. These discussions often centred on other unwell patients in the ward or another team needing the theatre team and space for an emergency operation. Although this type of distraction is minor, it often builds up over the course of the operation and has been shown to affect the focus and attention of the operating surgeon [4, 6, 14]. Perhaps, this observed negative association is due to the engagement of the surgeon and team on different, simultaneous tasks [4]. Other auditory distractions encompassed irrelevant discussions, environmental noise or background music which has been reported to be deleterious to surgical and overall team performance [4, 5, 15, 16]. Interestingly, communication that contributes towards team camaraderie remains undefined. Further work will need to be carried out to capture human factors that contributed positively rather than negatively towards theatre teamwork. However, our study with its emphasis on negative factors, has shown that cumulative distraction adds onto the perceived increased levels of stress during theatre, with further undermining of the theatre team’s performance [17].

In our study, we demonstrated that equipment-related distraction carried greater severity in both lung transplantation and cardiac surgery. Unsurprisingly, Aurora et al. has highlighted that equipment-related event is a key intra-operative stressor that significantly affects surgical performance [17]. Examples of equipment-related issues included missing needles, diathermy not working or breakdown of suction at critical moments of operation especially during anastomoses. The severity of equipment-related distraction often relates the difficulty in achieving immediate resolution, thereby resulting in delays during the procedure and surgical stress. In our study, all observed cases had the full WHO checklist as well as the ICECAP tool. Hence, this finding highlights the need to emphasize the equipment part of the WHO checklist in order to prevent any further disruption to the surgical work-flow. Highlighting a possible equipment-related distraction prior to the start of a surgical case grants us the invaluable opportunity to resolve a potential key stressor.

### Complications

Our study showed an association between greater distractions and worse post-surgical outcomes. These outcomes were chosen because they are surrogate markers for the level of mental focus, impact patients directly and are regularly used as healthcare measures for the success of an operation. All bleeding points need to be addressed prior to end of operation. If focus is affected, important bleeding sources may be missed. This is similar to anastomotic complications as sharp mental focus is required to execute optimal spacing to ensure anastomotic integrity. Length of ICU stay reflects general post-operative complications for both cardiac surgery and lung transplantation. Although the variety of our outcome measures are limited, they were representative of immediate post-op and late post-op complications.

Of interest, we observed that our surgeons in both lung transplant and cardiac surgery were only aware of 19.2–21.3% of the total number of distraction events. This poses a conundrum as the state of being ‘unaware’ could imply a complete focus at task at hand. However, we also demonstrated that greater complication rates were significantly correlated with a greater number of distraction events. This finding implies that the surgeon and the team’s focus has been affected by the distractions in the surgical work-flow but not acted upon as it was ‘subconscious’. The cumulative effect of minor events leading to significant errors with adverse outcomes has been highlighted in previous human factor studies [1, 8]. In order to counteract this, the situational awareness of the surgeon could be heightened to identify the distraction events, thus leading to a proper and definitive resolution of distraction events. A more consciously aware surgeon would be able to act to reduce the distraction, with a quick diagnosis of the problem and swift resolution to return to optimal surgical work-flow. The development of the non-technical skills of the surgeon (NOTSS) [18] such as situational awareness, theatre team management and communication skills are recognized as increasingly paramount to improving surgical outcomes [19]. For the  futures, NOTSS and ICECAP could be used together to identify, address and correct for problems within the surgical work-flow that is unit-specific.

### Study limitations

There are several limitations with this study. Firstly, the behaviour of the operating team may change over time due to the team recall exercise after each ICECAP case study. This has been widely described as the Hawthorne effect [20]. To reduce the ‘Hawthorne Effect’, the specific theatre team to the case being observed were not informed at the beginning or during the operation that they were being observed as part of the study ‘blinding’ protocol. However, for the purposes of general theatre communication and safety, they were informed via email that this study would be taking place not if and when in any theatre. This was akin to informing the entire theatre team that a clinical trial is taking place but they were not aware if it was specifically happening to their case.

Secondly, the severity of a distracting event is not weighted based on the timing of the event during the surgical work-flow but rather upon the delay of its resolution. To incorporate both within the capture tool and to refine the grading system of severity in order to prove causative links between specific events with outcome, this would require a much larger sample size to make any robust statistical conclusions. Thirdly, we cannot directly compare the results derived from lung transplantation and cardiac surgery as both types of surgeries are quite different, albeit performed in the same cardiothoracic unit by the same team. Although this is one of the larger studies documenting distractions within cardiac surgery and lung transplantation [11], we acknowledge all the limitations associated with a single-unit study and its own unique work culture. Of note, there is no background music as a unit policy to reduce background noise. Hence, a wider usage of this ICECAP capture tool in other units with different operating cultures could be conducted in order to further validate and refine the capture tool.

## Conclusions

ICECAP as a novel surgical distractions capture tool was effective and applicable for both elective cardiac and urgent transplant surgeries. Surgeons were unaware of a large number of distractions and interruptions whilst operating. Distractions were associated with longer ICU stay and higher rate of bleeding. Effective communication-fostering strategies (NOTSS) [18, 19] should be implemented in order to reduce distractions, improve teamwork and overall surgical performance. In order to effectively evaluate and refine ICECAP as a cardiothoracic distractions capture tool, it could be applied within other cardiothoracic units within the UK. If similar findings were replicated within other units, this would lend support to an acute need to address and subsequently, rectify our surgical work culture to improve surgical outcomes.

## Supplementary Information

**Additional file 1.** The Imperial College Error Capture Tool (ICECAP).

## Data Availability

Data from the study can be obtained via contacting Dr James Arkley via email: james.arkley@nhs.net.

## References

[1] Wiegmann DA, ElBardissi AW, Dearani JA (2007). Disruptions in surgical flow and their relationship to surgical errors: an exploratory investigation. Surgery.

[2] de Leval MR, Carthey J, Wright DJ, Farewell VT, Reason JT (2000). Human factors and cardiac surgery: a multicenter study. J Thorac Cardiovasc Surg.

[3] Mason SL (2013). Design and validation of an error capture tool for quality evaluation in the vascular and endovascular surgical theatre. Eur J Vasc Endovasc Surg.

[4] Wheelock A, Suliman A, Wharton R, Babu ED, Hull L, Vincent C, Sevdalis N, Arora S (2015). The impact of operating room distractions on stress, workload, and teamwork. Ann Surg.

[5] Mentis HM, Chellali A, Manser K, Cao CG, Schwaitzberg SDA systematic review of the effect of distraction on surgeon performance: directions for operating room policy and surgical training. *Surg Endosc.* 2015;30:1713–1724 10.1007/s00464-015-4443-z PMC5663645 26194261

[6] Sevdalis N, Undre S, McDermott J, Giddie J, Diner L (2014). Smith G Impact of intraoperative distractions on patient safety: a prospective descriptive study using validated instruments. World J Surg.

[7] Vincent C, Neale G, Woloshynowych M (2001). Adverse events in British hospitals: preliminary retrospective record review. Br Med J.

[8] ElBardissi AW, Wiegmann DA, Dearani JA, Daly RC (2007). Sundt TM 3rd Application of the human factors analysis and classification system methodology to the cardiovascular surgery operating room. Ann Thorac Surg.

[9] Healey AN, Primus CP (2007). Koutantji M Quantifying distraction and interruption in urological surgery. Qual Saf Health Care.

[10] Zheng B, Martinec DV, Cassera MA (2008). Swanström LL A quantitative study of disruption in the operating room during laparoscopic antireflux surgery. Surg Endosc.

[11] Gurses AP, Kim G, Martinez EA, Marsteller J, Bauer L, Lubomski LH, Pronovost PJ, Thompson D (2012). Identifying and categorising patient safety hazards in cardiovascular operating rooms using an interdisciplinary approach: a multisite study. BMJ Qual Saf.

[12] Antoniadis S, Passauer-Baierl S, Baschnegger H, Weigl M (2014). Identification and interference of intraoperative distractions and interruptions in operating rooms. J Surg Res.

[13] Martinez EA, Thompson DA, Errett NA, Kim GR, Bauer L, Lubomski LH, Gurses AP, Marsteller JA, Mohit B, Goeschel CA, Pronovost PJ (2011). Review article: high stakes and high risk: a focused qualitative review of hazards during cardiac surgery. Anesth Analg.

[14] Weigl M, Müller A, Vincent C, Angerer P, Sevdalis N (2012). The association of workflow interruptions and hospital doctors' workload: a prospective observational study. BMJ Qual Saf.

[15] Siu K-C, Suh IH, Mukherjee M, Oleynikov D (2010). Stergiou N The impact of environmental noise on robot-assisted laparoscopic surgical performance. Surgery.

[16] Way TJ, Long A, Weihing J, Ritchie R, Jones R, Bush M, Shinn JB (2013). Effect of noise on auditory processing in the operating room. J Am Coll Surg.

[17] Arora S, Sevdalis N, Nestel D, Woloshynowych M, Darzi A, Kneebone R (2010). The impact of stress on surgical performance: a systematic review of the literature. Surgery.

[18] Yule S, Flin R, Paterson-Brown S, Maran N. Non-technical skills for surgeons in the operating room: a review of the literature. *Surgery.* 2006; 139:140–9. Review. 10.1016/j.surg.2005.06.017 16455321

[19] Yule S, Paterson-Brown S (2012). Surgeons' non-technical skills. Surg Clin North Am.

[20] McCarney R, Warner J, Iliffe S, van Haselen R, Griffin M, Fisher P (2007). The Hawthorne effect: a randomised, controlled trial. BMC Med Res Methodol.

