# Supplementary figures and images for: A distractions capture tool for cardiac surgery and lung transplantation: impact on outcomes
Source: J Cardiothorac Surg. 2023 Jan 23;18:46. doi: 10.1186/s13019-022-02065-5 (PMC9872388; doi:10.1186/s13019-022-02065-5)

*Appendix 1*


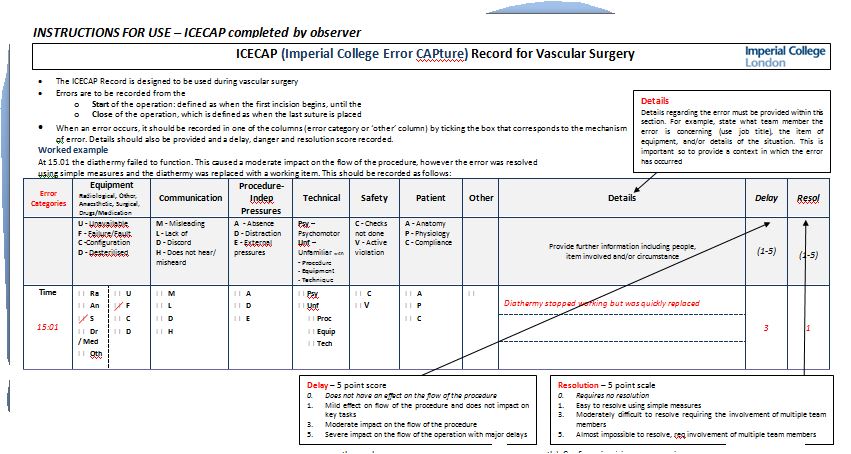


Figure 1: Explanation of ICECAP (Tool) Application


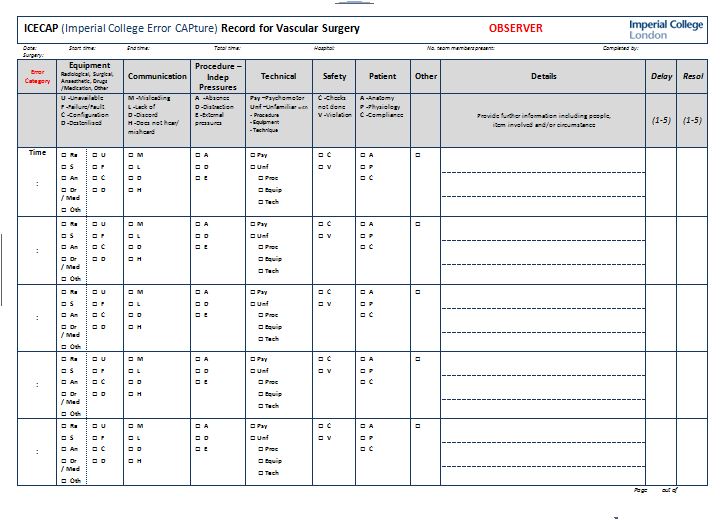


Figure 2: Full Outline of ICECAP Tool

Supplement: Supplementary file 1 — Additional file 1. The Imperial College Error Capture Tool (ICECAP). [file 13019_2022_2065_MOESM1_ESM.docx]
